# Towards Automated Inspections of Tunnels: A Review of Optical Inspections and Autonomous Assessment of Concrete Tunnel Linings

**DOI:** 10.3390/s23063189

**Published:** 2023-03-16

**Authors:** Andreas Sjölander, Valeria Belloni, Anders Ansell, Erik Nordström

**Affiliations:** 1 Division of Concrete Structures, KTH Royal Institute of Technology, Brinellvägen 23, 114 28 Stockholm, Sweden; 2 Geodesy and Geomatics Division, Department of Civil, Constructional and Environmental Engineering (DICEA), Sapienza University of Rome, 00184 Rome, Italy

**Keywords:** automation, mobile mapping systems, tunnel inspection, tunnel assessment, tunnel concrete damage

## Abstract

In recent decades, many cities have become densely populated due to increased urbanization, and the transportation infrastructure system has been heavily used. The downtime of important parts of the infrastructure, such as tunnels and bridges, seriously affects the transportation system’s efficiency. For this reason, a safe and reliable infrastructure network is necessary for the economic growth and functionality of cities. At the same time, the infrastructure is ageing in many countries, and continuous inspection and maintenance are necessary. Nowadays, detailed inspections of large infrastructure are almost exclusively performed by inspectors on site, which is both time-consuming and subject to human errors. However, the recent technological advancements in computer vision, artificial intelligence (AI), and robotics have opened up the possibilities of automated inspections. Today, semiautomatic systems such as drones and other mobile mapping systems are available to collect data and reconstruct 3D digital models of infrastructure. This significantly decreases the downtime of the infrastructure, but both damage detection and assessments of the structural condition are still manually performed, with a high impact on the efficiency and accuracy of the procedure. Ongoing research has shown that deep-learning methods, especially convolutional neural networks (CNNs) combined with other image processing techniques, can automatically detect cracks on concrete surfaces and measure their metrics (e.g., length and width). However, these techniques are still under investigation. Additionally, to use these data for automatically assessing the structure, a clear link between the metrics of the cracks and the structural condition must be established. This paper presents a review of the damage of tunnel concrete lining that is detectable with optical instruments. Thereafter, state-of-the-art autonomous tunnel inspection methods are presented with a focus on innovative mobile mapping systems for optimizing data collection. Finally, the paper presents an in-depth review of how the risk associated with cracks is assessed today in concrete tunnel lining.

## 1. Introduction

After decades of increased urbanization, many cities have become densely populated, and the transportation infrastructure has been heavily utilized. Concrete infrastructure such as tunnels, bridges, and roads often constitutes an important part of the infrastructure network. The downtime of these structures has a huge impact on the efficiency of transportation capacity; therefore, it is important to minimize this downtime to ensure cities’ functionality and economic growth. At the same time, the majority of concrete infrastructure is ageing, and continuous inspections are needed to ensure the safety of these structures. Concrete infrastructure can today be designed for a technical lifespan of up to 120 years, but it needs to be maintained to ensure safety. Inspections must be conducted to detect, observe, and measure damage such as cracks, water, and leaching, which can lead to decreased structural capacity. Cracks are a common initiation to the degradation of concrete and reinforcement, water often accelerates the deterioration, and leaching is common in structures with one-sided water pressure, such as tunnels [1,2]. Leaching is normally a slow process that takes many years to initiate, but the process can be accelerated if cracks are present or the quality of the shotcrete is lower. Thus, the extent of leaching in relation to when the tunnel was constructed may indicate low-quality shotcrete or cracks.

Nowadays, tunnels have become a more attractive alternative when road and railway networks need to be improved, and they have increased in both total length and number [3]. One of the main reasons for this is that a tunnel does not interfere with the existing city landscape. However, in transportation tunnels, there is widespread evidence of associated deterioration [4]. Thus, regular inspection and maintenance are vital to ensure their structural integrity over their entire lifespan. Today, on-site inspections of tunnels are almost exclusively performed by certified inspectors and/or structural engineers at regular intervals (routine inspection) through manual time-consuming activities [5]. The outcome of inspections usually depends on the engineers’ experience, which makes the assessment qualitative and subject to possible inaccuracies, as well as false or missing detection. In tunnels, inspectors are usually only equipped with a hammer, a head torch, and graduated cards provided with a set of different thickness lines normally used to visually estimate the crack width. For detailed inspections, a skylift is often required to be sufficiently close to the tunnel surface, which makes the inspection even more complex and time-consuming. Additionally, since the infrastructure must be closed during the inspection, these activities are normally carried out at night in a limited time interval to minimize the impact of tunnel downtime. This aspect, combined with the length/width of the infrastructure to monitor, makes it very difficult to inspect in detail, increasing the risk that potentially dangerous damage is not detected [6]. Moreover, tunnels are usually characterized by humidity, dust, and the absence of natural light and therefore represent an unfriendly environment for human activities [7]. Finally, tunnels are manually inspected over time by different operators, and knowledge must therefore be transferred between different inspectors and between inspectors and owners. This is complicated when only manually collected images and handwritten notes from different inspectors are available for monitoring.

**Figure 1:**
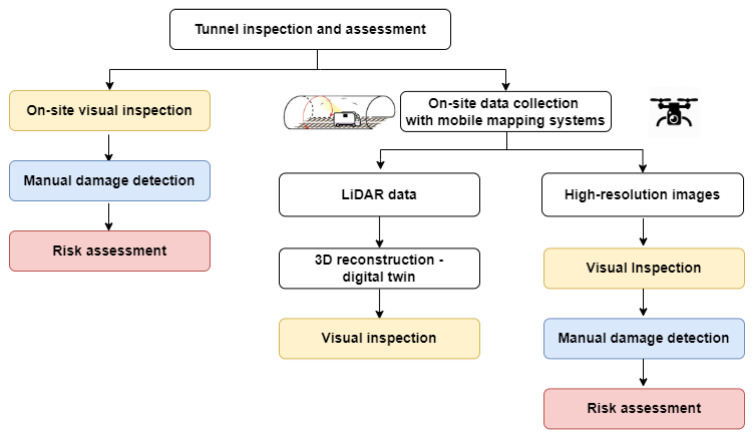
Standard procedure for tunnel inspection and assessment.

Automated, cost-effective, and exhaustive approaches for inspecting tunnels can, therefore, significantly increase the efficiency of inspections, improve the conditions for human activities, decrease the downtime of infrastructure and facilitate the transfer of knowledge [6]. In recent decades, efforts have been devoted to improving and automating inspection practices, but there is still a lack of knowledge in the field of assessment and maintenance of infrastructure. The introduction of mobile mapping systems with sets of geomatics sensors, i.e., visible and infrared (IR) cameras, light detection and ranging (LiDAR) sensors, inertial measurement unit (IMU), and distance measuring instrument (DMI), adopted for acquiring large amounts of data, has greatly improved the inspection protocol. This means reducing the time required, improving the quality of work, and increasing the amount of data available for measurements and maintenance [8]. In particular, high-resolution images and LiDAR data have been used to visually inspect infrastructure and reconstruct a 3D digital model (digital twin), enabling remote inspection from the offices and facilitating data and knowledge transfer. However, damage and structural condition detection is still manually performed on-site or in the office using the collected data, having a high impact on the efficiency and accuracy of the procedure (Figure 1).

The inspection is usually followed by an assessment of the structural condition. In this phase, the risk associated with the observed damage is evaluated with respect to the structural capacity and stability of the infrastructure. Commonly, these evaluations are used to classify infrastructure using condition classes adopted as the foundation for maintenance planning. If effective, the maintenance program helps reduce costs, decrease the number of temporary closures, increase public safety, and ensure adequate service levels. However, there are no internationally accepted standard procedures for conducting tunnel assessment and maintenance, which represents an open problem.

This paper presents a review of existing methods used to optimize data collection and inspection for tunnel monitoring and to assess the risk associated with damage in the concrete lining used as a rock support based on scientific papers and national guidelines. The paper is organized as follows: Section 2 gives a description of the damage and degradation of concrete in tunnels; Section 3 presents a review of autonomous tunnel inspection systems. Section 4 presents an overview of tunnel assessment procedures from scientific papers and national guidelines. Finally, in Section 5, some conclusions and future research are outlined.

## 2. Damage and Degradation of Concrete in Tunnels

Over time, damage to the concrete lining, such as cracking and leaching, that is visible is inevitable [9]. Cracks are formed for several reasons, such as drying shrinkage, temperature loading, structural overloading, or settlements. Due to low tensile strength, cracking is a natural part of the load-bearing mechanism for reinforced concrete. Therefore, cracks found during an inspection do not necessarily implicate an unsafe structure. Commonly, damaged sections initially have no or low impact on the structural capacity or the stability of the structure. However, a strong correlation exists between the width of the crack and the rate of various deterioration mechanisms such as carbonation and corrosion [10,11], which, over time, may have a significant effect on the safety of the structure. Therefore, damage should be detected early, and its progress over time should be monitored to increase the understanding of its effect on structural safety. This section presents different types of concrete tunnel linings and damage that can be detected during an optical inspection, along with typical causes and possible consequences. For a more thorough review of damage to tunnels and concrete structures, see, e.g., Strauss et al. [12] and the guidebook for tunnel inspections issued by the French Transport Administration [13].

### 2.1. Different Types of Linings

**Figure 2:**
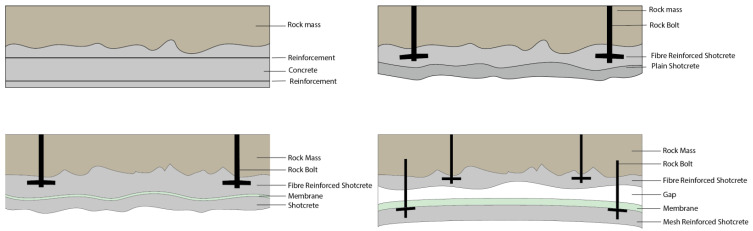
Different types of commonly used concrete rock support. From top to bottom, left to right, reinforced concrete, shotcrete on hard rock with rock bolts, sandwich shotcrete lining with a waterproof membrane, and shotcrete with a second waterproof lining, from Sjölander [14].

Reinforced concrete is the most commonly used support material for tunnels. Different types of support systems are used for tunnels depending on the strength and quality of the rock mass, alongside other factors, such as span length, type of tunnel, and height of overburdened rock mass. For a case with low-strength rock, it is commonly assumed that the support system must carry the entire weight of the rock mass. In such a case, an arch made of reinforced concrete is a typical support system. For a tunnel in good-quality rock, the rock mass can often support its weight through arch action; here, fiber-reinforced shotcrete, i.e., sprayed concrete, is often used in combination with rock bolts to support the tunnel. Shotcrete is directly sprayed on the rock surface and sticks to the surface through the use of set accelerators and high pneumatic pressure used during application. Depending on the tunnel requirements and the amount of infiltrating water, shotcrete can be complemented with different solutions to reduce the amount of water inside the tunnel. In Figure 2, two commonly used structural support systems are shown, together with two support systems used to reduce the infiltrating water.

It should be noted that the post cracking response significantly varies between fiber-reinforced and conventional reinforced shotcrete, which should be considered in the assessment, see, e.g., Sjölander [14]. Moreover, damage severity mainly depends on the type of lining. Conventional reinforced concrete and shotcrete directly sprayed on the rock are both parts of the primary support system and have a structural purpose, while shotcrete in a secondary lining is part of a functional lining that should prevent the ingress of water in the tunnel. This must also be considered in the evaluation of the risk associated with the crack.

### 2.2. Cracks in the Concrete Lining

When concrete cracks, the stiffness of the material locally decreases. As mentioned, cracks do not necessarily have a direct impact on the structural capacity of the tunnel lining. The impact of the crack with respect to the structural capacity depends on the length and width of the crack and on factors such as the direction of the crack with respect to the tunnel. Moreover, the cause of cracking has a significant effect on its impact, and the structural post cracking performance of the concrete lining must also be considered. Cast reinforced concrete has strain-hardening behavior, meaning the structure can carry an increased load after cracking. Fiber-reinforced shotcrete, on the other hand, has normally a strain-softening behavior, which implies that the load-carrying capacity decreases after the first crack is formed [14]. This is true for a structure with no horizontal restraint, e.g., a simply supported beam [15,16]. A tunnel lining is normally confined, either through in situ compressive stress caused by the deadweight of the rock mass or by restrained movement owing to the interaction between shotcrete and rock. This partly restrains the cracks from opening and may change the behavior from strain-softening to strain-hardening, see, e.g., Nilsson [17].

If possible, cracks should be divided into two categories to facilitate the assessment of the associated risk:

- Load-independent cracks caused by, e.g., restrained shrinkage or swelling of the concrete;
- Load-induced cracks caused by, e.g., loose blocks or deformation of the tunnel.

Nordström et al. [18] also suggested that cracks can be divided into categories based on the time of failure, i.e., before or after hardening. With only optical data (e.g., visible and IR images) and LiDAR acquisitions available, such a task is difficult to automate and will require human expertise and interaction.

#### 2.2.1. Load-Independent Cracks

For concrete lining, load-independent cracks, caused before or after hardening, are commonly found in tunnels. Ansell [19] reported more than 900 cracks during an in situ mapping before the opening of a tunnel, and Malmgren et al. [20] mapped the fall-out of shotcrete along the drift of a mine in northern Sweden. In both cases, drying shrinkage was the most likely cause of the failures. Such cracks are formed when the movement of the concrete is restrained by, e.g., the bond to the rock. Once a crack is formed, the stress is released, and the cracks are normally stable, i.e., no propagation is expected over time. For a cast reinforced concrete lining and fiber-reinforced shotcrete lining with a continuous bond to the rock surface, a systematic pattern with narrow cracks is expected, while few and wide cracks are expected for a shotcrete lining in which the bond between shotcrete and rock is partially lost [21,22]. Shrinkage cracks are commonly oriented perpendicular to the tunnel axis with a systematic distance between them. However, as shown by Sjölander and Ansell [22], slight variations in the crack pattern should be expected for linings with local variations in shotcrete thickness. One more common cause of early concrete cracking is temperature variations. Hydration is an exothermic process in which the concrete elongates. At an early stage, the concrete is plastic, and elongation can normally occur without any build-up of stresses. During cooling, the material has hardened, and the shrinkage is internally restrained, which results in a build-up of stresses and possibly cracking. For shrinkage cracks, temperature cracks normally occur perpendicular to the direction of the tunnel and with a systematic distance between them. For carefully designed concrete, a fine pattern of narrow cracks should form, which reduces the risk associated with the cracks.

Shrinkage and other load-independent cracks can often be considered harmless [23]; however, over time, shrinkage cracks can initiate deterioration, such as corrosion of the reinforcement and internal spalling due to freezing. An evaluation of the residual flexural strength of cracked steel fiber-reinforced shotcrete after 17 years of field exposure in various environments was presented by Nordström [24]. Here, it was shown that the crack width was important in determining the time for initiation of corrosion but was less important for the corrosion rate. Most samples with an initial crack width of 0.5 mm or more showed a significant loss in residual strength, up to 60% after 17 years of exposure. Hence, cracks should be repaired early to avoid such degradation of the structural capacity occurring over time.

#### 2.2.2. Load-Induced Cracks

**Figure 3:**
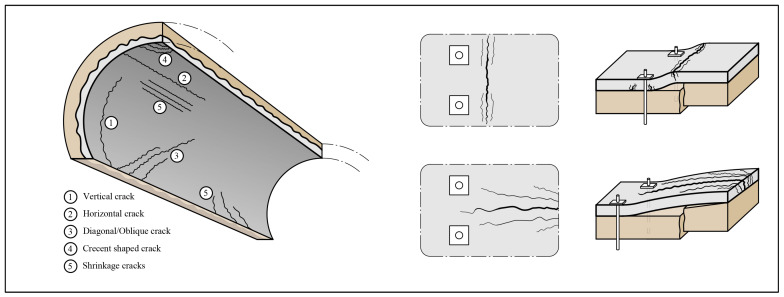
Different orientations of structural cracks and possible causes [23] and possible crack patterns for concrete lining subjected to the load from a loose block [25,26,27].

Typically, load-induced cracks originate from the loads caused by the deformation of the rock or soil, water pressure, or the deadweight of a loose block. Here, the French guideline [23] makes an effort to distinguish between horizontal, vertical, and diagonal structural cracks as well as crescent-shaped cracks and how they should be assessed for cast reinforced concrete linings, see Figure 3.

Horizontal cracks are orientated parallel to the tunnel direction, and several horizontal cracks commonly occur in succession. Single horizontal cracks are uncommon. The risk associated with cracking is low, but the formation of such cracks may decrease the stiffness and cause the localized rupture of a lining. However, as long as no rapid deformation occurs at these sections, it is suggested that no actions should be taken [23]. Vertical cracks caused by loading are normally wider than the vertical cracks caused by shrinkage. A common cause of the cracks is variations in the concrete thickness and differential settlements of the surrounding rock or soil mass [23]. For horizontal cracks, no immediate actions need to be taken unless signs of rapid deformation are shown. The same recommendations are also given for diagonal cracks. However, this type of crack can indicate more severe problems for the tunnel on a global scale, such as instability or foundation settlements. Therefore, it might be wise to follow up on this type of crack and investigate its possible cause. Crescent-shaped cracks normally start and end in segmental cast stages. The guideline [23] suggests that these are formed during cracking and do not influence the structure’s safety.

Based on experimental testing by Holmgren [25], Fernandez-Delgado [26], and Andersson [27], possible crack patterns caused by the loading from a loose block are shown to the right in Figure 3. Here, the tests by [25,26] are shown at the top and were static tests performed on a fiber-reinforced shotcrete lining with 2D behavior. These crack patterns likely develop in bolt-anchored and fiber-reinforced shotcrete lining with low horizontal in situ stresses subjected to a static load. At the bottom right in Figure 3, crack patterns from the test by Andersson [27] are shown. Here, a conventional reinforced concrete lining was subjected to impact loading. These crack patterns are, thus, representative of a secondary lining subjected to the dynamic loading from a loose block.

### 2.3. Influence of Cracks

**Figure 4:**
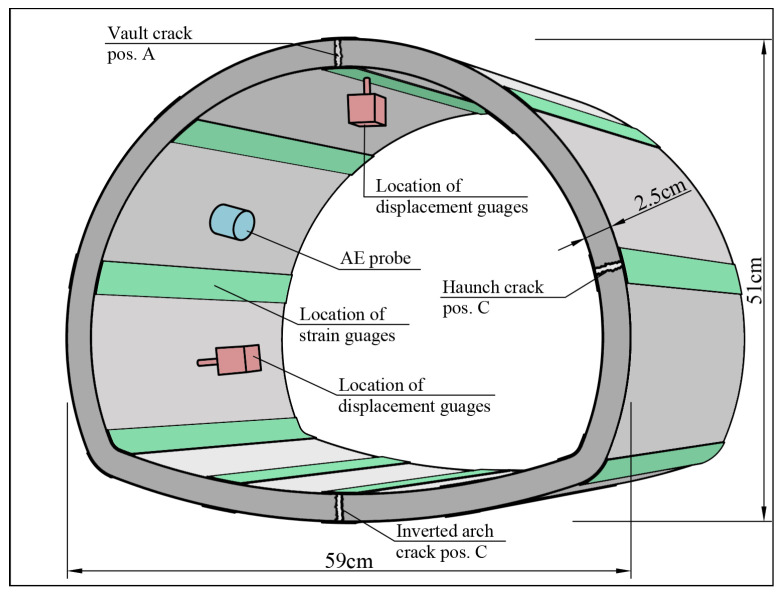
Experimental set-up and location of existing cracks in testing performed by Su et al. [28].

Most guidelines on how cracks influence the structural capacity of concrete lining are based on empirical knowledge, but few scientific studies have been devoted to the risk of cracks in tunnel linings. However, Su et al. [28] investigated how pre-existing cracks affect the structural capacity of an unreinforced concrete ring by performing 1:10-scaled tests in a laboratory environment. The tunnel section was subjected to uniform pressure, and cracks with a 600 × 0.2 × 10 mm (length × width × depth) were introduced in the model before loading. Every model contained one crack, and the failure load was investigated using four different crack locations and compared with an uncracked model, see Figure 4.

Here, the results showed a decreased structural capacity of approximately 5% when a crack was present. This result indicates that the direct influence from narrow cracks with a limited depth is negligible with respect to the structural capacity. Xu et al. [29] combined scaled physical tests and numerical simulations using the finite element method (FEM) to investigate how the presence of cracks affects the mechanical behavior of concrete lining. The existing cracks had a depth equal to half of the model thickness and ran across the entire model width. The experiments showed that the internal force in a section close to existing cracks significantly decreased while the displacement in the same section increased. Through numerical simulations, it was shown that existing cracks far apart did not influence each other, and their effects could be treated separately. When cracks appeared closer together, a coupling effect existed between the cracks, which should be accounted for [29].

### 2.4. Infiltrating Water

In a tunnel, the primary rock support is in direct contact with the rock mass and is therefore affected by the groundwater. Because a dry traffic environment is desirable, preventive actions, such as grouting or watertight membranes, are adopted to reduce the contact between groundwater and rock support. However, such systems are rarely completely successful, and part of the tunnel and the concrete is therefore subjected to a one-sided hydro-static pressure. This can lead to erosion or an accelerated leaching process, which is discussed further below. Infiltrating water in the tunnel is a sign of lost water tightness or failure of installed drains in the tunnel. Over time, the water pressure, in combination with cracks, may lead to internal erosion. This loss in material volume is strongly correlated to a reduced strength of the material [30]. A more severe risk that includes water is freezing. Because ice occupies a larger volume than water, the volume expansion caused by freezing can lead to spalling of the concrete.

During construction, water may be present on the rock surface. This can result in a low-quality or nonexisting bond between concrete and rock [31]. Thus, partial wet sections can indicate a section with a poor bond. Because the water transportation in uncracked concrete is mainly governed by a slow diffusion process, local wet sections can also indicate the presence of cracks or more porous concrete. Based on the results from an in situ inspection, Feng et al. [32] suggested that extensive cracking in tunnel walls is due to long-term exposure to water that alters the mechanical properties of the rock mass. The softening of the rock mass leads to increased pressure on the rock support, which results in cracking.

### 2.5. Leaching of Concrete

**Figure 5:**
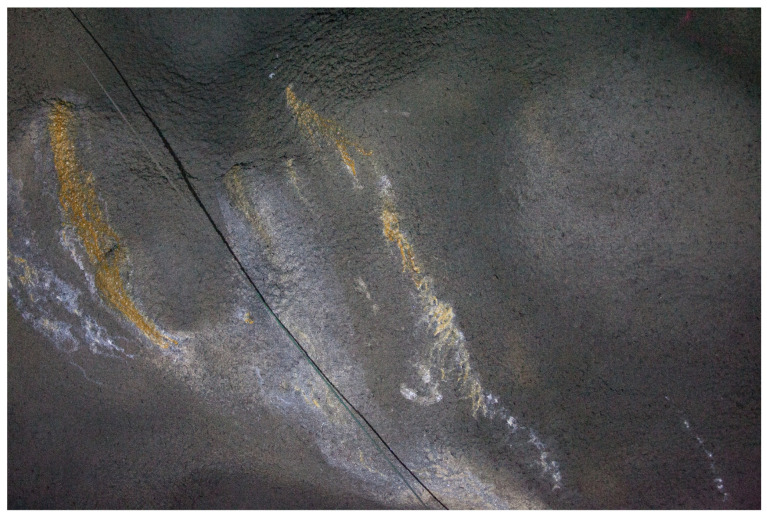
Examples of leaching of the shotcrete in a hard rock tunnel, from Sjölander and Ansell [1].

During the leaching process, calcium hydroxide (CH) is dissolved in water and transported to the concrete surface through a slow diffusion process or with the flow of water, which is a much faster process. When particles of CH react with the carbon dioxide (CO${}_{2}$) on the surface of the concrete, precipitation is formed. This is normally white but can also be colored depending on the content of the dissolved product; see examples in Figure 5.

In an in situ investigation performed by Sjölander and Ansell [1], early leaching and the presence of cracks in a shotcrete rock support were investigated. No visible cracks were found, and most leached areas were dry, indicating that the leaching process had stopped. Likely, leaching was caused by one-sided water pressure in combination with early formed cracks. Under certain conditions, the transportation of dissolved CH can initiate the self-healing of existing cracks [9,33]. Because this stops the flow of water through the concrete, the leaching process is driven by diffusion, which significantly decreases the speed of the deterioration process. If leaching progresses over time, the material’s porosity increases, leading to a decrease in strength [2]. To summarize, leaching is a common deterioration phenomenon in tunnels; but, under most circumstances, it has a limited effect on the structural capacity. If leaching is ongoing for an extended period, the internal erosion of CH increases the porosity and decreases the strength. A sign of ongoing leaching progress is an increased area with precipitation or a wet area.

### 2.6. Nondetectable Damage Types

Above, the defects that can be detected with an optical inspection system, i.e., cracks, infiltrating water, and leaching, were presented. However, several other types of damage exist that cannot be detected with an optical system. Such damage types must therefore be detected by in situ inspections or other methods such as ground penetration radar, ultrasound, or by striking the surface of the concrete with a hammer. Different types of damage that must be detected by other methods are:

- Voids/bond failure between concrete and rock;
- Internal erosion;
- Corrosion of steel.

Cracks, leaching, or infiltrating water may be either an indication or an initiation of the above damage types. Therefore, an in situ inspection of some of these areas should be considered a good practice, even though the visible damage alone may not directly impact the tunnel’s structural safety.

## 3. Autonomous Tunnel Inspection

Technological advancements in the areas of computer vision, AI, and robotics have recently opened up the possibility of the automated inspection of tunnels. In the last decades, several companies and research projects have investigated the use of robotic and mobile platforms to improve tunnel inspection procedures.

### 3.1. Mobile Mapping Systems’ Data Collection

**Figure 6:**
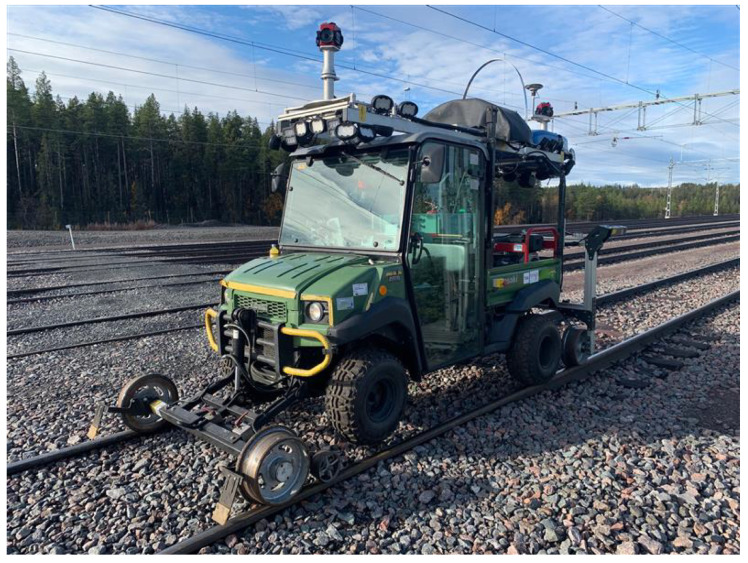
Mobile mapping system for tunnel inspections developed by the company WSP [38].

Thanks to the wide availability of low-cost sensors, advances in computational resources, and the maturity of mapping algorithms, many industries have started looking into the possibilities of automating and increasing the efficiency of monitoring systems [34]. All over the world, many companies have developed mobile mapping systems with different sensors. Today, such companies install geomatics sensors on vehicles or drones to design mobile mapping systems that automatically collect different types of data from the surrounding environment. The mobile mapping solution Leica Pegasus:Two is among the most adopted mobile mapping systems and uses laser scanners in combination with GNSS receivers, an IMU, a DMI, and cameras installed on the vehicle to acquire accurate spatial data [35]. The recently updated version of Pegasus:Two (Pegasus:Two Ultimate) removes the need for six-camera stitching by incorporating two back-to-back cameras, creating a 24 MP 360-degree image calibrated to the LiDAR profiler data. The Trimble MX9 system combines long-range LiDAR, spherical imagery, and oblique cameras along with inertial sensors for capturing dense point clouds and both panoramic and multiangle images [36]. The Topcon IP-S3 HD1 mobile mapping system integrates an IMU, a GNSS receiver, a vehicle odometer, LiDAR sensors, and a six-lens digital camera system. The mobile mapping system offers high-density and high-precision point clouds combined with high-resolution panoramas [37]. For tunnel infrastructure inspections, the companies WSP, ETS s.r.l., Euroconsult and Pavemetric, and Omnicom Balfour Beatty have developed customized mobile vehicles to maximize the use of combined sensors in the complex environment of tunnels. The WSP system contains six LiDAR scanners, two panoramic cameras, nine high-resolution IR cameras, and IR flashes adapted to illuminating tunnel lining (Figure 6).

This technology is periodically adopted for tunnel monitoring and maintenance in combination with GeoTracker software [38]. Moreover, the potential for using deep-learning techniques for damage detection using this mobile mapping equipment was investigated [6,39]. The ARCHITA system, developed by ETS s.r.l, is a multidimensional system that combines different devices, including the Leica Pegasus:Two and Tunnel Scan systems. It consists of various devices: laser scanners, georadars, and thermal and high-definition digital cameras. It is a customized rail mobile mapping system able to provide a series of information: georeferenced 3D point clouds, thickness and status of the infrastructure together with cavities and alterations, and crack and water detection from high-resolution images and thermographic analysis. The mapping of defects is carried out by combining the high-resolution images taken by the three high-definition cameras of the Tunnel Scan system and the point cloud acquired with laser scanner technology. In theory, the combination of the two technologies makes the images measurable, with the possibility of locating, measuring, and quantifying the defects identified on the tunnel surface [40]. Euroconsult and Pavemetrics developed a tunnel inspection system based on up to six cameras with laser line illumination that allows for scanning tunnel wall linings. They also provide software for the Tunnelings system, which allows the data from two different inspections to be easily compared and structural changes and all types of wall lining defects to be assessed [41]. Finally, the Digital Imaging for Condition Asset Management (DIFCAM) system, developed by Omnicom Balfour Beatty, records high-resolution images of tunnel linings and is paired with precise positioning, LiDAR scanning, and autonomous digital image correlation (DIC) software capable of automatically detecting significant changes in the external aspect of the tunnel lining [42,43,44]. DIC is a very powerful technique at the laboratory scale, see, e.g., [45,46,47], but it presents some limitations outside laboratory conditions that strongly limit its application.

In the last decades, different research projects have developed innovative and customized platforms for data collection in tunnels. Yu et al. [48] proposed a small mobile robot equipped with a charged-coupled device (CCD) camera to inspect and measure cracks in concrete surfaces through computer vision algorithms. Yao et al. [49] developed and investigated a similar system equipped with ultrasonic sensors and video cameras. The inspection consisted of a scan of the tunnel lining in order to search for deformations. Gavilan et al. [41] presented a tunnel inspection system developed by Euroconsult and Pavemetrics. The system is based on cameras and laser sensors that allow the scanning of a tunnel lining at speeds up to 30 km/h. Victores et al. [50] proposed a robot-aided system to inspect and maintain weakened surfaces of roadway tunnels with minimum interference with passing traffic. The ROBO-SPECT European FP7 project [7] proposed a robotized system for structural tunnel inspection designed for roadway tunnel monitoring. Huang et al. [51] designed a subway tunnel image capture system based on CCD line-scan cameras to collect metro tunnel surface data. They proposed an algorithm based on the local image grid features to recognize cracks and the Otsu algorithm to detect leakages. Finally, Li et al. [52] presented an automatic Metro Tunnel Surface Inspection System (MTSIS). They devised a data collection system to capture tunnel surface images with high resolution, and they presented a tunnel surface image preprocessing approach and a damage detection method to recognize defects using deep learning approaches. Due to the speed of mobile mapping systems, the tunnel must, in most cases, be closed in order to securely and accurately collect data. This is related to the frequency of data collection of the adopted sensors. When the speed of the mobile mapping system is high, there is less time to collect data. Therefore, for LiDAR sensors, more laser beams are needed to ensure that enough points are measured to reconstruct the object of interest [53]. For collecting images, high-frequency cameras should be used. Mett et al. [54,55] presented interesting results: a high-speed camera was mounted on a vehicle to collect imagery at a speed of up to 50 km/h. In this way, data could be collected while the tunnel was open.

### 3.2. Mobile Mapping System Data Processing

**Figure 7:**
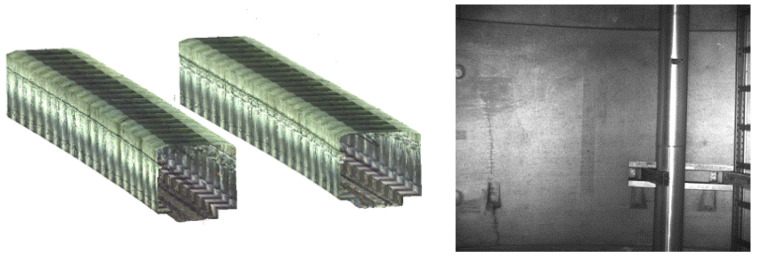
Tunnel data collected using WSP mobile mapping systems: LiDAR point cloud reconstruction (**left**) and high-resolution image (**right**).

In the case of long infrastructure systems such as tunnels, bridges, and roads, the data collected using mobile mapping systems can provide different information including a 3D model of the structure, the so-called digital twin, and allow its visual inspection to find cracks and other types of damage. In tunnels, inspections are usually carried out by combining LiDAR point clouds and high-resolution images (Figure 7).

The 3D reconstructions from LiDAR technology mounted on mobile mapping systems have become a standard procedure for tunnel monitoring thanks to the capability of these instruments to collect 3D coordinates of millions of points, which can reproduce the scanned surface with a good level of detail and allow for a virtual inspection of the infrastructure. Moreover, if the resolution and tolerance of the sensors are greater than the magnitude of the displacements, 3D measurements can be performed by comparing the scanned cavity geometry at various times [12,56]. Additionally, the acquired high-resolution images can be used to visually and accurately inspect the infrastructure and find damage. Moreover, through state-of-the-art photogrammetric software, e.g., Agisoft Metashape, Pix4D, Context Capture, and Leica Infinity, high-resolution images can be processed to generate accurate dense point clouds and 3D models. This approach is particularly useful for 3D reconstructions when unmanned aerial vehicles (UAVs) are used to collect data of infrastructure such as bridges, see, e.g., Mirzazade [57].

**Figure 8:**
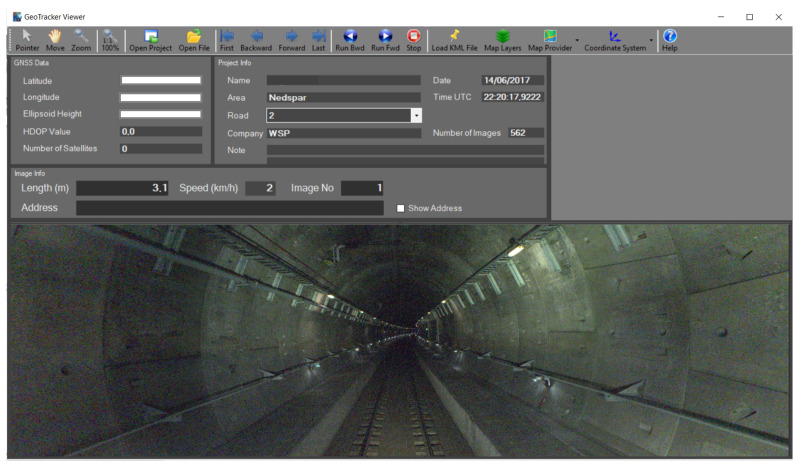
Geotracker software interface used by the company WSP.

Even if mobile mapping systems enable the acquisition of large amounts of data and the 3D reconstruction of the infrastructure allows for global inspections and displacement measurements of a certain magnitude, damage detection is not always possible due to the limited resolution of the reconstructions. Furthermore, there are still no official procedures commonly adopted by monitoring and maintenance companies for automatic damage detection. This means that the majority of inspections are still visually performed on the collected data (with a focus on the high-resolution images) using specific software packages such as the Mobile Mapping GeoTracker, which only facilitate data visualization (see Figure 8). The operation is, therefore, still time-consuming and subjected to human errors.

In the last decades, different image-based approaches have been widely investigated to overcome all these drawbacks and to develop an automatic procedure for damage detection. The aim is to speed up the procedure of infrastructure monitoring and achieve the efficient automatic extraction of defects. Some examples are edge-detection algorithms, percolation methods, and principal component analysis (PCA), see, e.g., [58,59,60]. Recently, interesting results have been achieved thanks to the investigation of advanced deep learning approaches. Among them, convolutional neural networks (CNNs) have proven to be effective approaches for automatic damage detection (image classification, object detection, and semantic segmentation) [6,61,62,63,64,65]. With the increased interest from the scientific community in using CNNs to automatically detect concrete damage, various prelabeled datasets are now available for training deep learning architecture and evaluating their potential [66,67,68,69,70,71]. However, these techniques are still under investigation, and the level of maturity is still not sufficient for real applications. This is the reason why, until now, these techniques have not been adopted by maintenance companies worldwide, especially because the task of defect detection represents a crucial aspect of inspection with a high impact on the assessment of the infrastructure, as explained in the following section.

## 4. Assessment of the Safety of Damaged Concrete Linings

Commonly, the assessment of damaged concrete lining is divided into local and global effects. First, local effects, i.e., individual damages, are separately assessed. This means that the impact of each damage is first assessed. This should be performed with respect to the structural capacity and the prescribed functionality of the structural part. For example, a crack in a waterproof membrane likely does not affect the structural stability but may significantly reduce the functionality. Second, the tunnel is divided into sections, and the structural condition and functionality are assessed based on the combined effect of all local damage in that section.

### 4.1. Damage Diagnostics

Most of the published papers on maintenance focus on developing autonomous inspection and damage detection methods. Farrar and Lieven [72] expressed that damage diagnostics and structural condition assessment remain elusive tasks. In many standards and national guidelines [23,73,74], damage diagnostics are periodic with a fixed interval. However, Ai et al. [75] suggested that periodic inspections are problematic because the interval is subjectively decided with no theoretical basis. Moreover, the deterioration rate during the structure’s lifetime varies, which should be reflected in the choice of inspection interval.

In its essence, damage diagnostics and the subsequent planning of required maintenance is an optimization problem that must find a sufficient balance between the cost of maintenance and the required level of safety of the tunnel. Different strategies to optimize maintenance are presented in the literature, see, e.g., Ai and Yuan [76], Baji et al. [77], and Zuluaga and Sánches-Silva [78]. Regardless of the methodology used, the problem is determining suitable threshold values for the observed damage to decide if any countermeasures need to be taken or if the damaged section does not impact the structural stability or safety and can be ignored. To optimize this work, Lara et al. [79] suggested that the assessment of the structure should be divided into stages in which the quality of the data and the decision increase for every step. Moreover, Bien et al. [80] suggested that three levels of damage diagnostics should be performed. The first step should focus on damage detection, the second on damage diagnostics, and the third on identifying simulators and catalyzes of the degradation process. To optimize the surveillance and monitoring of a concrete dam, Nordström et al. [81] used a risk matrix to classify the need for monitoring in each dam section.

### 4.2. Autonomous Tunnel Assessment

To the best of our knowledge, only a few studies and research projects focusing on autonomous tunnel assessment of concrete tunnels exist. In the literature, a quantification of tunnel lining level of degradation was proposed using the Tunnel Lining Crack Index (TCI) [82]. The TCI was introduced because the severity of cracks in the lining highly influences the mechanical stability of a tunnel. It is based on crack tensor theory, see, e.g., [83,84], and considers the width, length, and direction of cracks on the lining surface, allowing the judging of the crack maximum length and width, distributions, and directions. According to the authors, the health assessment system based on TCI (applied in Japan) is reasonable with some limitations. Indeed, the complex distribution of cracks makes the crack intersection (not considered in the TCI) prevalent, which can greatly influence the stability of a tunnel lining [85]. For this reason, further research has been carried out, and alternative assessment methods have been investigated to provide a more comprehensive assessment of tunnel stability. Jiang et al. [86] proposed a quantitative inspection and assessment system for tunnel linings that is capable of acquiring and detecting defects and assessing the condition of tunnels using the fractal dimension D index. In fractal geometry, fractal dimension analysis provides a statistical index of complexity that can explain how the fractal pattern changes with the measuring scale. The authors verified the applicability of the proposed inspection system on two tunnels built with different construction methods [86]. In the study, they used the index to evaluate the condition of the lining according to the fractal dimension of cracks. Then, they compared it with the TCI to verify its performance. Some researchers in the framework of the IN2TRACK project have also been investigating and developing inspection strategies and maintenance systems in tunnels. These research projects are based on the idea of life cycle management, which is a complex field involving engineering, management, and information technology. In such research, the aim is to develop a maintenance management system composed of a basic information module, inspection module, analysis module, and maintenance module that interact with a building information model (BIM) database to support the inspection [42,87].

From a commercial point of view, a few companies have recently investigated new approaches for tunnel assessment. Among them, ETS s.r.l. developed the ETS management and identification of the risk for existing tunnels (MIRET) approach, which combines the ARCHITA system with AI technologies to detect defects from the high-resolution images [88,89]. MIRET is a methodology for the integration of ARCHITA data and the digital design and management of tunnels. The process combines innovative mobile mapping survey systems, defect analysis, AI, and risk analysis. The purpose of MIRET is to develop a management approach using a series of procedures, both on large and small scales, which define the existing risk in tunnels. The system defines a unique risk value for the tunnel and the sectors that make it up. The information is then integrated into an infrastructure management protocol. The risk definition also provides an index to perform maintenance and inspection [88]. Finally, Network Rail designed a system called the Tunnel Condition Marking Index (TCMI) which measures the change in tunnel stock condition over time. The system can be applied to brick, stone, and concrete-lined tunnels (excluding segmental concrete) and provides a quantitative condition mark for each tunnel after an examination. During the inspection, each occurrence of damage is collected and a code is assigned to it taking into account its type and dimensions. Then, an algorithm associates a score for each minor element (i.e., crown or sidewall). The condition mark grades the structure on a scale of 0 to 100, where 100 is assigned to a tunnel in perfect condition and is based on a nonjudgemental recording of defects by the examiner. Scores lower than 40 indicate bad conditions and scores greater than 80 indicate good conditions. TCMI scores are generated at minor, major, or tunnel bore levels. The condition of each element is recorded as an alphanumeric severity/extent code assigned to the defect during the inspection. These codes include information on the type, area or length, and number of defects and are the input to algorithms able to provide a TCMI score [42].

### 4.3. National Guidelines for Assessment

Performing a quantitative assessment of damage to concrete structures has not received much attention from the scientific community, and few papers focus on how to assess individual damages. However, some countries have established national guidelines to increase the quality of inspections and obtain more consistent results from their inspectors. A summary of some of these guidelines is presented below.

In Sweden, road and railway tunnels on the public transportation network are divided into a functionality class that ranges from TK0 to TK3 [74]. Here, the former implies a tunnel section with no apparent risk of lost functionality within ten years, while the latter indicates signs of lost functionality during the inspection and requires immediate countermeasures. It is important to note that lost functionality does not necessarily mean the tunnel’s safety is affected. During an inspection, individual damage is individually assessed. Then, each tunnel section is classified based on the sum of individual damage occurrences. An individual point system is available to guide the inspector in assessing individual damages. In Table 1, the grading system to assess the severity of individual cracks in concrete/shotcrete lining that is part of a structural support system is given [74]. Here, it should be noted that this is used as guidance for unreinforced and fiber-reinforced shotcrete as well as conventional reinforced concrete. Hence, no distinguishing is made between the different types of concrete linings. As guidance for assessing a cracked section, the guideline states that individual cracks rated as level 4 could belong to TK3. However, the engineering judgement of the inspector should also be used to assess the section.

The French Road Administration has issued a guideline for tunnel inspection [23] and a library of tunnel damage [13]. A systematic approach to inspect and assess the condition is presented in [23]. The tunnel is divided into fixed, homogeneous sections in terms of structural and geotechnical context. Then, each structural part in every section is classified and given a “Civil Engineering Rating” and a “Water Rating”. Parts are then classified according to the IQOA system defined by the French State. A summary of the deterioration level for the different condition classes used for the Civil Engineering Rating is given in Table 2. For cracks, there are no specific guidelines to correlate the crack geometry to a specific IQOA rating. According to the guideline, cracks belong to condition class 1 to 3U [23]. To assess the structural condition and plan the maintenance, each section is divided into smaller areas that have similar IQOA values.

Similar to the French guidelines [23], a definition and summary of different types of damage commonly found in tunnels are presented in a Tunnel Maintenance Manual [90]. No specific guidelines are, however, given on how damage and the presence of cracks should be evaluated. Sound engineering judgement should be used to evaluate the risk associated with damage, the consequences of failure, and the cost of proposed maintenance. However, in a guideline issued by the Massachusetts Department of Transportation [73], a clear guideline on how to correlate the length and width of cracks to a condition class is defined; see Table 3.

In Table 4, the assessment criteria for cracks in tunnel linings based on the Chinese standard [91] is presented. Here, grade 1A indicates that structural damage may affect the safety of the users, i.e., pedestrians and vehicles, and that countermeasures should be taken. A similar system is also available to assess the severity of leaking water.

As shown above, different national guidelines differently assess the risk associated with cracks. The main reason for this is that the support of tunnels is designed differently depending on the quality of the rock mass and the expected load on the support. Thus, a risk system should be developed for every type of tunnel support. The structural capacity and risk with respect to cracks in reinforced cast concrete have been well studied, see, e.g., [92,93,94]. However, for fiber-reinforced concrete, there is a lack of research on experimental testing and numerical simulation focusing on the effect of cracks.

## 5. Conclusions and Future Research

This paper reviewed the methods and technologies for inspecting and assessing concrete tunnels. Scientific papers and commercial solutions for improving the objectivity and accuracy of tunnel monitoring were presented. Additionally, scientific papers and national guidelines for tunnel assessment were reviewed and presented in detail in this paper. Based on this review, the following conclusions can be drawn:

- In recent years, robotic and mobile mapping systems have been developed for automatically acquiring data in tunnels and improving the inspection protocol. This technology is mature and already exists on the market.
- Damage detection is still mainly performed manually, which has a high impact on the efficiency and accuracy of the procedure. However, the interest from the scientific community in developing and improving deep learning algorithms is constantly improving the efficiency and accuracy of damage detection.
- Some indications, such as preferred orientation and common crack widths, exist for load-induced and load-independent cracks. However, it can be concluded that the cause of cracking is difficult to determine in an automated way on the first inspection. Over time, cracks caused by shrinkage or temperature, i.e., load-independent cracks, tend to be stable. Hence, the cause of cracking can be better estimated after several inspections. The experimental tests reported in the literature indicated that cracks with limited width (here, less than 0.2 mm) have no direct impact on the structural capacity of the concrete lining.
- Different methodologies to assess the safety of the tunnel and the need for maintenance are, nowadays, adopted together with recommended threshold values for assessing the risk associated with cracks in concrete lining based on national standards. It can be noted that a significant difference exists between the national guidelines. The threshold values contain a combination of numeric values and descriptive text. This makes the guidelines difficult to use in an automated process in which only numeric values can be used. Furthermore, neither theoretical reasoning nor experimental testing is provided to give any scientific background to the selected threshold values for cracks presented in the guidelines. Thus, the values are most likely determined based on a combination of empirical knowledge and subjective engineering judgement. To increase the understanding of the risk associated with cracks in the concrete lining, numerical simulations or more experimental testing is one possible way forward.

To summarize the current state of research, it seems likely that automated inspections and assessments of tunnels will be implemented in the coming years. This review showed that the technology to efficiently and semiautonomously capture data from a tunnel is mature and, to some extent, already available on the market. Moreover, the efficiency and accuracy of deep learning algorithms to automatically detect cracks and other defects are constantly improving. Here, further research should focus on proof of concept on a large scale, i.e., the accuracy of detecting cracks in different tunnels and environments must be shown. The results should not only be compared with the ground truth, i.e., labeled data, but also with the results from human in situ inspection. This way, the accuracy and efficiency of automated and human inspections can be compared. Lastly, a foundation for an automated assessment system must be developed, and threshold values for cracks and other damages must be suggested. In this area, more research is required.

## Figures and Tables

**Table 1:** Summary of important descriptions for the assessment of damage levels for cracks in shotcrete and concrete in interaction with rock, from guidelines by the Swedish Transport Administration [74].

Level	Description
1	Web pattern of cracks with diameter less than 50 cm.
2	Crack with a length smaller than the block size.
3	Cracks larger than the block size or web-pattern cracks with a diameter larger than 50 cm.
4	Cracks larger and longer than 1 m and/or follow the join patterns in the rock mass.
5	Cracks with a width and length larger than 1 mm and 1 m, respectively.

**Table 2:** Summary of Civil Engineering Rating based on the IQOA system from the French guidelines [23].

Class	Description of Deterioration in Area
1	In good visual condition.
2	Minor deterioration that does not affect the stability of the structure.
2E	Same as class 2 but higher possibility of continuous degradation and/or increase in extent.
3	Deterioration indicates that the structure has been altered or that the stability might be affected.
3U	Indicates of deep/severe damage that affects the overall stability.
S	The indicator “S" can be included in any of the classes and indicates possible danger to the user.

**Table 3:** Summary of important descriptions for the assessment of cracks in concrete roof slab and walls from guidelines by the Massachusetts Department of Transportation [73].

Class	Description
1	Width less than 0.3 mm or spacing greater than 1.5 m.
2	Width between 0.3 to 2.5 mm or spacing between 0.3 to 1.5 m.
3	Width greater than 2.5 mm or spacing less than 0.3 m.
4	The effect with respect to structural stability and capacity must be evaluated in detail.

**Table 4:** Grading of cracks according to the Chinese standards from [91].

Structure	Crack Width	Crack Length	Grade
Lining	≤3 mm	≤5 m	1A
Lining	≤3 mm	>5 m	1A
Lining	>3 mm	≤5 m	2A
Lining	>3 mm	>5 m	2A/3A

## Data Availability

No research data available.
